# Hazelnut Skin in Ewes’ Diet: Effects on Colostrum Immunoglobulin G and Passive Transfer of Immunity to the Lambs

**DOI:** 10.3390/ani12223220

**Published:** 2022-11-21

**Authors:** Irene Viola, Paolo Tizzani, Giovanni Perona, Carola Lussiana, Antonio Mimosi, Patrizia Ponzio, Paolo Cornale

**Affiliations:** 1 Department of Veterinary Science, University of Turin, Largo Paolo Braccini 2, 10095 Grugliasco, TO, Italy; 2 Department of Agricultural, Forest and Food Sciences, University of Turin, Largo Paolo Braccini 2, 10095 Grugliasco, TO, Italy

**Keywords:** sheep, colostrum, welfare, immunity, antioxidants, hazelnut skin

## Abstract

**Simple Summary:**

A ewe’s diet in the last period of gestation can modify the immunoglobulin G composition of the colostrum and consequently the health status of lambs. This study aims to determine the role of hazelnut skin on the immunological colostrum quality and the passive immunity transfer in newborns. The results show that hazelnut skin supplementation in the diet positively affects the immunoglobulin G composition of the colostrum. The experimental trial underlines that the use of by-products in livestock feeding provides a paramount opportunity to create a circular economy system with health benefit on farmed animals.

**Abstract:**

Passive immunity transfer has a pivotal role in newborn lambs, where the colostrum represents the primary source of immunoglobulins. This study hypothesized that the high content in polyphenolic compounds, mono- and poly-unsaturated fatty acids, and vitamin E of hazelnut skin affects blood and colostrum immunoglobulin G (IgG) concentration and related gamma-glutamyl-transferase (GGT) and lactate dehydrogenase (LDH) levels in sheep and their lambs. In the last 45 days of pregnancy, ewes were divided into a control (CTR) and a hazelnut skin supplemented group (HZN). Blood and colostrum were collected from ewes and lambs before the first suckling, at 24 and 48 h after birth, then IgG concentration, GGT and LDH activity levels were measured. IgG concentration in the colostrum and in lamb’s serum were significantly greater in HZN than CTR. No significant difference was detected for ewe’s blood. A significant positive correlation was found between IgG and GGT in lambs’ serum and colostrum, between IgG and LDH, as well as between GGT and LDH in lambs’ serum and colostrum. Our results suggest that hazelnut skin supplementation influences IgG colostrum concentration, with improved immune passive transfer to the suckling lambs. The transfer of maternal derived immune factors is confirmed by the GGT and LDH enzyme activity levels.

## 1. Introduction

Sheep farming for meat production represents a significant economic opportunity for the livestock sector. The lambs of meat sheep breeds (e.g., Bergamasca, Merino, Suffolk, Ile de France) are exclusively fed with maternal milk until weaning at 60–90 days of life. As a result, they are characterized by higher birth weight and better growth performances than dairy sheep lambs. In addition, the natural suckling lamb feeding system ensures better animal welfare and meat quality compared to the artificial milk feeding regime [1]. The lambs’ immune system activity depends primarily on the quality of the ewe’s colostrum [2,3].

The acquisition of passive immunity by early ingestion of maternal immunoglobulins from the colostrum is critical for newborn survival. The ingestion of the colostrum should occur within the first 12–24 h after birth, as intestinal absorption of the immunoglobulins rapidly decreases [4,5]. Inadequate colostrum intake or a poor-quality colostrum may cause the failure of passive immunity, with increased morbidity and mortality rates [6,7].

The immunoglobulins G (IgG) are the primary markers for evaluating passive immunity transfer to newborns [8,9,10]. Therefore, IgG concentration defines the immunological quality of the colostrum [11,12]. IgG concentration is quantified both in the colostrum and in the lamb’s blood serum in the first 24–48 h after birth, in order to evaluate the antibody coverage [13]. IgG concentration in lamb’s blood serum should exceed 15 g/L within 24 h from delivery, to minimize natimortality risk due to neonatal infections [14,15]. Significant differences in the immunological quality of the colostrum are reported not only among ruminant species, but also between sheep and goat breeds: dairy sheep have lower colostral IgG levels than meat sheep [16,17].

Several studies have investigated the relationship between colostrum-mediated immunity and the expression of specific blood serum enzymes, such as gamma-glutamyl-transferase (GGT) and lactate dehydrogenase (LDH) [18,19,20,21]. The colostrum and the blood’s enzymatic activity are indirect indicators of passive immunity transfer [20,21,22]. The GGT catalyzes the transfer of amino acids in the protein synthesis process and is mainly expressed in tissues with intense secretive and absorbed activity, including the mammary gland [23,24,25]. Increased GGT expression is related to the high levels of colostral IgG and milk protein [26,27]. The LDH is considered an indicator of mastitis in cows, since its activity levels have turned out to be positively correlated with IgG concentration in milk [28]. Currently, GGT is considered the most reliable marker of antibody coverage, and a positive correlation between GGT and IgG has been demonstrated in cows, sheep, and goats [29,30,31], whereas the relationship between LDH and other blood serum and colostrum parameters is still sparsely investigated [32,33,34].

The quantity and quality of the colostrum is influenced by the health status and diet of ewes during the gestational period [35]. Some authors highlighted the relevance of dietary conjugated linoleic acid, tocopherols, and polyphenols in reducing oxidative stress and increasing the antibody levels, thus contributing to maintaining an effective immune response [36,37,38].

Current evidence shows that by-products of fruit and vegetable processing can be used in farm animal nutrition as functional feed ingredients to improve food product quality [39]. The use of agricultural residues has positive economic and environmental effects throughout the entire supply chain [40]. In addition, most of these by-products represent a source of phytochemicals (carotenoids, phenolics, and flavonoids), antioxidants, antimicrobials, vitamins, and dietary fatty acids (FA) with favourable nutritional properties [41]. The positive effects of hazelnut consumption, related to their content in polyphenolic compounds, mono- and poly-unsaturated FA, and vitamin E, are reported in the literature [42]. Recent studies also note the beneficial effect of the hazelnut skin integration in ruminant feeding due to oleic acid content and antioxidant effects [43,44,45].

This study aimed to evaluate the effect of hazelnut skin on the immunological colostrum quality and immunity status of Biellese ewes and lambs during the *post-partum* period. Specifically, this work analysed IgG, GGT and LDH levels in both the serum and colostrum of ewes and lambs following different diets (with and without hazelnut skin inclusion). The second objective was to investigate the relationship between IgG concentration and GGT and LDH activity levels in blood and colostrum to assess their use as predictors of passive immunity.

## 2. Materials and Methods

### 2.1. Etichal Approval

The project was approved by the Italian Ministry of Health, General Directorate of Animal Health and Veterinary Pharmaceuticals, Office VI (n°493/2020-PR, 15/05/2020).

### 2.2. Animal Management

The study was carried out at the Teaching Farm of the Department of Veterinary Science (University of Turin, Italy). Twenty multiparous Biellese ewes at second and third parity, aged 18–36 months, with an average live weight of 75–80 kg and body conditions score (BCS) equal to 2.75–3.25 were estrus-synchronized using 60 mg medroxyprogesterone acetate intravaginal sponges (Ovigest, Hipra, Spain) for 14 days, followed by an intramuscular injection of 125 µg cloprostenol sodium (Estrumate, MSD, Madison, NJ, USA) at sponge removal [46,47]. The ewes were mated with two rams (1:10 ewes) 48 h after sponge removal. Pregnancy was confirmed by ultrasound scanning at day 45 after mating (Esaote MyLab One, ultrasound probe 5–10 MHz, Genova, Italy) and the ewes were randomly assigned to two homogeneous groups (10 animals/group) based on their weight, age, and BCS. The dams were housed in two 20 m^2^ (2 m^2^/animal) pens and kept under standard management and environmental conditions, in compliance with the European Directive (Council Directive 98/58/EC) on the minimum standards for the protection of animals bred or kept for farming purposes. Animal health status was monitored throughout the experimental period. Only singleton delivery sheep were selected. Sheep with clinical symptoms in the peri-partum period were excluded from the study. At the end of the lambing time, each group included eight ewes and eight lambs.

### 2.3. Dietary Treatments

The experimental diets were formulated following NRC recommendations [48]. During the last 45 days of pregnancy, the ewes of the control group (CTR) were daily fed 1.2 kg of mixed hay and 0.5 kg of an experimental pelleted concentrate containing (%): barley (36.0), maize (36.0), soybean meal (18.1), and rumen-protected fat (5.4) (Magnapac^®^, Norel & Nature Nutrition—Madrid, Spain). The hazelnut skin group (HZN) received 1.0 kg of the same mixed hay and 0.5 kg of a second experimental pelleted concentrate, in which the rumen protected fat was replaced by hazelnut skin. This byproduct was collected from a local producer (Az. Agr. Durando—Portacomaro, AT, Italy) and it was obtained from the kernel’s separation after the roasting process. The concentrate contained (%): barley (27.3), maize (27.3), hazelnut skin (27.3), and soybean meal (13.6). Both experimental concentrates also included (%): calcium carbonate (2.0), sodium chloride (1.0), precipitated dicalcium phosphate dihydrate (0.5), sodium bicarbonate (0.3), magnesium oxide (0.2), and mineral-vitamin premix (0.5). The proximate compositions of experimental feedstuffs are presented in Table S1. The two experimental diets were isonitrogenous (116 and 121 g/kg DM for the CTR and HZN diets, respectively) and isoenergetic (6.1 and 6.3 MJ/kg DM for the CTR and HZN diets, respectively) (Table 1). During the lactation period, the hay was increased from 1.2 to 1.6 kg/day and from 1.0 to 1.4 kg/day in the CTR and HZN groups, respectively, to cover feeding requirements. Consequently, energy and protein concentration were also comparable in the two diets for the lactation period (Table 1).

The experimental concentrates and hay were offered individually, since all ewes were kept at individual feeders, twice a day (at 8.00 a.m. and 4.00 p.m.). The established amounts of hay and concentrate were weighed before being distributed and were completely consumed by all of the animals.

### 2.4. Feeding Intake, Sampling, and Analysis

Feed ingredients were representatively sampled and stored until analysis. The samples were ground with a cutting mill to pass a 1-mm screen sieve (Pulverisette 15-Fritsch GmbH, Idar-Oberstein, Germany). AOAC procedures were used to determine dry matter (DM), ash, crude protein (CP), acid detergent fiber, and acid detergent lignin (ADF and ADL) [49]. Ether extract (EE) was analyzed according to [50]. The Van Soest et al. [51] method was used to determine neutral detergent fiber (NDF); α-amylase (Sigma Aldrich, Saint Louis, MO, USA), but no sodium sulphite was added and results were corrected for residual ash content. Rumen-degradable protein (RDP) was analyzed according to Licitra et al. [52]. The energetic value of feeds was expressed as net energy for lactation (NE_L_) and was estimated according to National Research Council equations [53].

The FA composition of feedstuffs was assessed using a combined direct transesterification and solid-phase extraction method as described by Alves et al. [54]. A detailed description of the separation, identification, and quantification of fatty acid methyl esters (FAME) is available in Cornale et al. [55].

FA daily intake (g/head) of individuals and groups was estimated considering the daily dry matter intake (DMI) and the analytically determined FA composition of each feedstuff (Table 2).

The contents of total extractable phenols (TEP), non-tannin phenols (NTP) and condensed tannins (CT) in hazelnut skin were determined according to Iussig et al. [56]. Total tannins (TT) were computed as the difference between TEP and NTP. Hydrolysable tannins (HT) were estimated as the difference between TT and CT [57]. The amount of phenolic compounds ingested by the ewes belonging to the HZN group was estimated based on the analyzed phenolic composition of hazelnut skin and the intake of the HZN concentrate.

All analyses were performed in duplicate.

### 2.5. Blood and Colostrum Sample Collection and Analyses

Newborn lambs were allowed to be licked and dried by their dams and then the lambs were weighed, sexed, and ear-tagged within 1 h after birth. Blood samples were collected in ewes and newborn lambs before suckling at birth (A), and then at 24 and 48 h after birth (B and C, respectively). Lambs were also blood sampled at 10 days after birth (D). Blood samples were collected by jugular vein-puncture and transferred into a 9-mL blood tube (FLmedical, Gel + Clot Act, Vacuumed). Blood samples were maintained at room temperature for 30 min for blood clotting and then centrifuged at 1500× *g* for 10 min. The serum was transferred into Eppendorf tubes and frozen at −20 °C until analysis.

Colostrum samples were obtained within 1 (A), 24 (B), and 48 h (C) after parturition. Samples were collected by hand-milking into 20-mL plastic tubes 10-mL from each udder, inverted 8–10 times to mix for thorough and accurate homogenization, and stored at −20 °C until analysis.

Serum and colostrum samples were labelled with the animal identification number and date of collection and transferred to the “Istituto Zooprofilattico Sperimentale della Lombardia e dell’Emilia ermediate layer was aRomagna”(Brescia, Italy). Colostrum samples were thawed and centrifuged at 4000× *g* for 15 min to remove fat and sediments. The supernatant was then centrifuged at 18,000× *g* for 1 h and the intermediate layer was analysed [32]. Serum samples were thawed at room temperature 1 h before laboratory processing.

Blood serum electrophoresis was carried out with a HYDRAGEL 30 Beta1-Beta2 protein electrophoresis kit (Sebia, Issy Les Moulineaux, France) using the HYDRASIS and PHORESIS software (Sebia, Issy Les Moulineaux, France) [58,59].

Colostrum total protein was determined using the biuret method [60]. Protein fractions were determined by electrophoresis separation using the HYDRAGEL protein kit (Sebia, Issy Les Moulineaux France) and quantified using a densimeter (CGA, Florence, Italy).

The GGT and LDH activity levels (Szazs/Persijn Method, g-GT LIQUID; D.G.K.C. method, LDH-P) were determined by a biochemical automatic analyzer IL650 (Instrumentation Laboratory, Lexington, MA, USA).

### 2.6. Data Analysis

All statistical analyses were performed using R software version 4.0.3 (R Core Team 2020). Differences in dry matter, fatty acids and polyphenols intake between the two experimental diets were analyzed using a mixed model for repeated measures over time considering the effect of dietary treatment, the effect of sampling date, and their interaction. Results are reported as LSMeans.

A descriptive analysis of IgG, LDH, and GGT concentration in serum and colostrum was calculated using means and standard deviation. The differences in IgG, LDH, and GGT by the CTRL and HZN group were graphically evaluated through strip charts, using the ggplot R package [61]. A multilevel model was built to assess the effects of hazelnut integration (dietary treatment’s effect) on serum and colostrum IgG levels. The statistical model also included the fixed effect of sampling time, the random effect of ewes, and the fixed effect of the interaction between dietary treatment and sampling time. A hierarchical analysis was performed with R version 4.0.3 (R Core Team 2020). The lme4 v1.1-20 [62] and the lmerTest v3.1-0 [63] packages were used. The Akaike’s Information Criterion (AIC) [64] and the likelihood ratio test were used to select the best model. Homogeneity of variance was assessed with diagnostic plots from lattice v0.20-35 [65]. Significant differences were considered at *p* < 0.05.

A correlation analysis was carried out to evaluate the relationship among IgG, LDH, and GGT concentration in serum and colostrum, using the PerformanceAnalytics R package (https://CRAN.R-project.org/package=PerformanceAnalytics; accessed on 4 May 2021).

## 3. Results

### 3.1. DM and Fatty Acids Intake

The effects of dietary hazelnut skin in ewes’ diet on DM and FA intake during gestation and lactation periods are shown in Table 2. As already reported, concentrate and hay were always totally ingested in both experimental groups during the different periods. The DMI was comparable in the two groups and the differences were not significant either in the gestation or the lactation periods (Table 2). The analysis showed highly significant differences (*p* < 0.001) between the two groups of ewes in the two periods for the intake of all individuals and groups of FA from the diets. In particular, the HZN group showed a higher intake of monounsaturated FA (MUFA: +261% and +256% in gestation and lactation, respectively) and polyunsaturated FA (PUFA: +16% and +15% in gestation and lactation, respectively), as well as lower intakes of saturated FA (SFA: −43% and −40% in gestation and lactation, respectively) when compared to the CTR group.

The amounts of TEP, NTP, TT, CT, and HT ingested daily by the ewes of the HZN group were equal to 48.07, 19.03, 28.24, 3.29, and 24.94 g/head × day, respectively (data not reported in the table).

The interaction between dietary treatment and sampling date (DT × SD) was not significant for the considered parameters.

### 3.2. IgG Concentration

IgG concentration (g/L) in sheep and lamb’s blood and in the colostrum at each sampling time are reported in Table S2.

**Figure 1:**
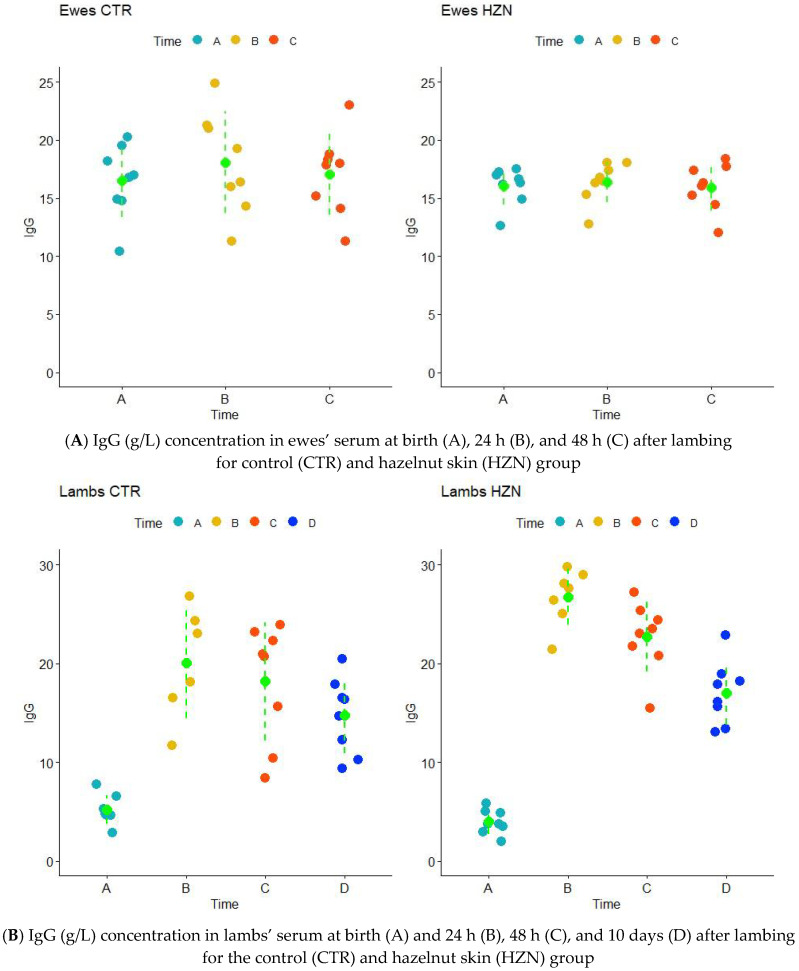

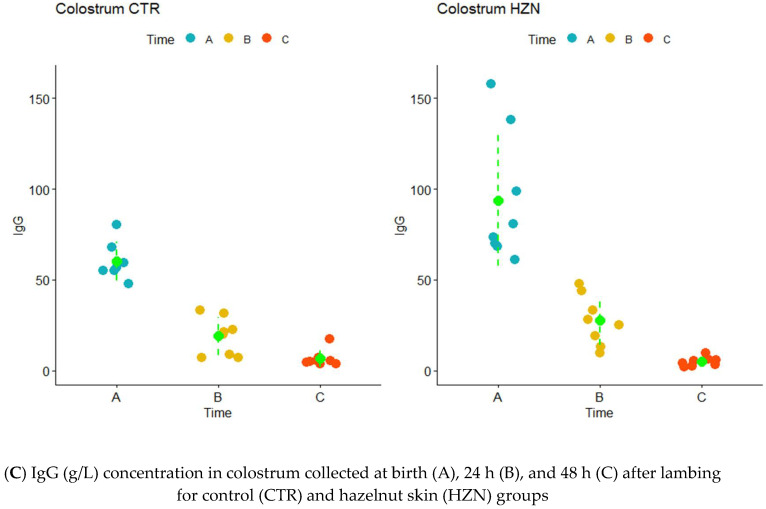
(**A**) IgG (g/L) concentration in ewes’ serum at birth (A), 24 h (B), and 48 h (C) after lambing for control (CTR) and hazelnut skin (HZN) group. (**B**) IgG (g/L) concentration in lambs’ serum at birth (A) and 24 h (B), 48 h (C), and 10 days (D) after lambing for the control (CTR) and hazelnut skin (HZN) group. (**C**) IgG (g/L) concentration in colostrum collected at birth (A), 24 h (B), and 48 h (C) after lambing for control (CTR) and hazelnut skin (HZN) groups.

The trend of IgG concentration in sheep was constant across sampling times in both groups (Figure 1A). However, IgG levels were more variable in the CTR group. The coefficients of variation (CV, %) of IgG in ewes of the CTR group were always higher than for the HZN group: 1.9-fold higher in time A (19.1% vs. 10.0%), 2.3-fold higher in time B (24.2% vs. 10.6%), and 1.6-fold higher in time C (20.6% vs. 13.0%). Furthermore, a larger portion of IgG data in the HZN group fell in the optimal range (15–18 g/L). However, according to the linear mixed effect model (LMM), the difference between the two groups was not significant (Table 3).

The pattern of IgG concentrations in lambs’ serum is comparable between the two groups (Figure 1B). The lowest IgG value was detected at birth, when newborns were almost lacking in antibody coverage [18]. Although IgG in lamb’s serum were slightly lower in the HZN group at birth, IgG then reached higher values compared to CTR samples. After 24 h of natural suckling, the IgG level speeds up to 22.8 ± 6.9 and 27.3 ± 3.0 g/L in the CTR and HZN groups, respectively. The IgG colostrum in HZN showed a higher frequency of large values (above 20 g/L) than the CTR group. In total, 94% of the lambs had IgG concentrations > 15 g/L, which is considered to be an indication of high passive immune transfer [15]. IgG concentration range at 24 h was from a minimum of 11.7 g/L in the CTR group to a maximum of 31.4 g/L for HZN lambs. The IgG level then slowly decreased: after 10 days, lambs reached an IgG concentration equal to 17.0 ± 3.2 and 14.7 ± 3.8 g/L in the HZN and CTR group, respectively. According to the mixed model, there was a significant “group effect” on IgG, with the HZN group showing higher values, with a very good fit of the model (Table 3).

Considering the colostrum, IgG concentration was clustered at lower values for the CTR group, while in HZN it was more variable but more frequent at higher values (Figure 1C). At lambing, the IgG level in the colostrum was very high (60.4 ± 10.7 g/L CTR; 94.0 ± 35.9 g/L HZN) and then steeply decreased to the lowest levels (6.6 ± 4.4 g/L CTR; 4.8 ± 2.4 g/L HZN). Also, in this case, the mixed model showed a significant effect of the group on IgG levels, with higher values observed in the HZN group, with a very good fit of the model (Table 3).

### 3.3. GGT and LDH Activity Levels IgG Concentration

Regarding the enzymes, the GGT activity level was equal to 50 ± 13 UI/L in the CTR group and 42 ± 16 UI/L in the HZN one, while the LDH activity level was 1077 ± 166 UI/L and 1132 ± 182 UI/L in CTR and HZN ewes, respectively. In the colostrum, GGT concentration was around 10,533 ± 1103 UI/L in the CTR group and 10,291 ± 1275 UI/L in the HZN one, while LDH was 973 ± 213 UI/L and 1024 ± 195 UI/L in CTR and HZN group, respectively. In the newborns, the GGT activity level was highly variable with 755 ± 970 UI/L and 1076 ± 1517 UI/L in the CTR and HZN group. Finally, mean ± SD lamb’s LDH activity level was 1503 ± 412 UI/L and 1403 ± 362 UI/L in CTR and HZN newborns.

### 3.4. IgG, GGT, and LDH Enzyme Activity Levels Correlation

**Figure 2:**
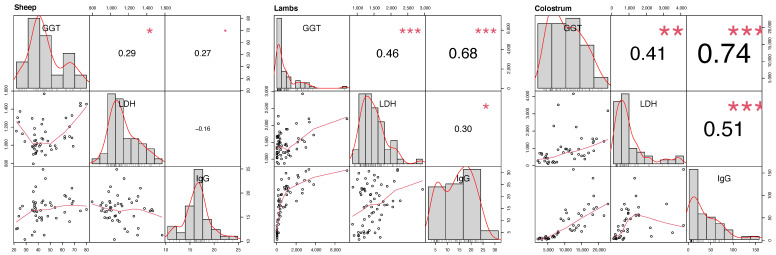
Correlation-chart IgG (g/L), GGT (UI/L), and LDH (UI/L) concentration in sheep and lambs blood serum and colostrum. The correlation value is represented for each pair of correlations. The asterisk indicates the significance level: <0.001 (***), <0.01 (**), <0.05 (*). The frequency distribution for each variable is represented by grey histograms, and their correlation by the plots on the left side of each group of figures.

No correlation was found between IgG and the analyzed enzymes in ewes’ serum; only a minimal correlation was found between GGT and LDH (R = 0.29; *p* < 0.05). A strong positive correlation was detected between IgG concentration and GGT activity level in lambs (R = 0.68; *p* < 0.001), while a minimal correlation was found between IgG and LDH (R = 0.30; *p* < 0.05). GGT and LDH also had a moderate correlation in newborns (R = 0.46, *p* < 0.001). A significant correlation was also detected between IgG concentration and GGT activity level in the colostrum (R = 0.74; *p* < 0.001), whereas the GGT-LDH and IgG-LDH correlation was moderate (R = 0.41; *p* < 0.01, and R = 0.51; *p* < 0.001) (Figure 2).

## 4. Discussion

To the best of our knowledge, the present study assessed for the first time the effect of hazelnut skin integration in sheep diet on the immunological quality of colostrum and passive immunity transfer to newborns. The results showed that the inclusion of hazelnut skin in the ewes’ diet during the peri-partum period significantly increased the IgG concentration of the colostrum and lamb’s serum. The IgG concentration was significantly correlated with GGT and LDH activity levels.

Thanks to the FA composition of hazelnut skin, the HZN ewes ingested higher amounts of MUFA (mostly C18:1*c*9) and PUFA, such as C18:2n6 (one of the precursors of conjugated linoleic acid-CLA). In a previous study [45] it has been demonstrated that the inclusion of hazelnut skin in a cow’s diet affects the FA profile of milk, increasing, among others, the CLA content. The same study also showed that hazelnut skin provides tocopherols and phenolic compounds. Although tocopherols have not been determined in the present study, it is reasonable to assume that HZN ewes obtained higher amounts of tocopherols and polyphenols than CTR ewes.

The antioxidant role of CLA, tocopherols, and polyphenols on the immunity system have been deeply investigated in the literature among the farm animals. Dietary CLA are antioxidants, able to reduce the lipid peroxidation and to protect the cell from oxidative damage by inducing de novo synthesis of glutathione in bovine mammary gland cells [36]. Similarly, α-tocopherol is a potent antioxidant and a free radical scavenger.

Several authors [66] showed that vitamin E supplementation supports ruminant health by enhancing cell-mediated and humoral immune responses, including all immunoglobulin classes [67]. Bondo and Jensen [68] demonstrated that a daily supplementation of α-tocopherol in the last 4 weeks of gestation increases IgM and IgG levels in a mare’s colostrum. IgG concentrations were higher in colostrum following alpha-tocopherol supplementation in the last gestational period in sows [69]. According to Żarczyńska et al. [70], the administration of selenium and α-tocopherol to cows during late pregnancy enhanced passive immunity transfer from the mother to the offspring, with higher IgG blood concentration in newborns. Moreover, the action of natural α-tocopherol on the immune system functionality was considerably higher compared to the synthetic vitamin E supplementation in ruminants [71]. On the contrary, Sterndale et al. [72] reported that maternal vitamin E supplementation during the late gestation does not influence IgG concentration in the colostrum, with no effect on the adaptive immune status of lambs.

Finally, the role of polyphenols in livestock nutrition is still a developing field, and their biological functions are being investigated more and more [38]. Studies have shown that phenolic compounds act as antioxidants by protecting tocopherols from oxidation and therefore increasing the tocopherols’ content in tissues [73]. Similarly, polyphenols preserve PUFA, rumenic and vaccenic acid from a complete biohydrogenation in the rumen, thus contributing to increasing the polyunsaturated portion and reducing the saturated content in milk [74]. Tannins have been reported to be involved in the regulation of the immune system in monogastric animals. Dietary tannins at appropriate dosages can positively affect immune system of chickens [75] and pigs [76]. Conversely, the potential role of supplemental tannins on humoral response in ruminants is sparsely explored and requires further investigations. Recently, Prodanović et al. [77] observed higher IgG levels in the colostrum of cows supplemented with chestnut tannins extract compared to control groups. These authors hypothesized that dietary tannins, depressing the protein degradation rate in the rumen, increased the protein portion available in the intestine, thus promoting colostrum quality and colostral IgG levels.

Vela [78] and Alves et al. [15] have shown that colostral IgG concentration is extremely variable in sheep, and huge differences between dairy and meat sheep breeds have been observed. In particular, previous studies reported an average colostral IgG concentration of 20.2 ± 8.0 g/L in Lacaune [17] and 31.3 ± 12.7 g/L in Bergamasca sheep [78]. Considering the average IgG levels reported in the literature among meat sheep breeds, the colostrum IgG concentration observed in our study for both groups was quite high; the HZN group in particular has a much greater mean value, reaching Merino’s IgG level (44.2 g/L) [17]. In the present study, the IgG concentration in colostrum significantly differed between the CTR and HZN groups. Despite the great variability of the IgG concentration in the colostrum, ewes fed a HZN diet showed a higher colostral IgG content than the CTR group (42.0 vs. 34.4 g/L, respectively; *p* < 0.05). Considering that the two groups were balanced, and the experimental design has been carried out to minimize other confounding factors (such as farming environment, health management, and animal genetics), the difference in the colostral IgG concentration could suggest an effect of the inclusion of hazelnut skin in the diet, considering its tocopherols and phenolic compounds.

In the current study, IgG concentration > 15 g/L was recorded in all the lambs; this value is considered an indicator of efficient passive immune transfer [14,15]. In both groups the IgG concentration in newborns was similar to the concentration of healthy adult sheep (15.0 to 17.0 g/L) [79]. It is well-known that immunity status in ruminant newborns is solely dependent on the intestinal uptake of the immunoglobulin and other bioactive proteins [80], and the ewe’s diet influences the colostrum composition [35]. The results underlined that the diet affects IgG concentration more in the colostrum than in the ewe’s blood serum, a specifically higher IgG concentration was detected in HZN lambs due to the best IgG composition of the colostrum. The IgG concentration in ewes’ serum was similar in each group. Our data agree with the results of Vatankhan [81] that detected values ranging from 15.9 to 21.3 g/L in adult sheep. No differences emerged between CTR and HZN IgG concentration in ewe’s serum, indicating that a pre-partum evaluation of the IgG level in ewe’s blood is not predictive of the colostrum’s immunological quality. In agreement with the literature, the IgG quantification in newborn’s blood within 48 h after birth is the best method for measuring the passive immunity transfer [11,12].

The evaluation of GGT and LDH enzyme activity both in sheep’s serum and colostrum showed interesting results. GGT activity levels were found to agree with the literature [79]. On the contrary, LDH activity levels in our work were higher than previous findings (230–400 UI/L) [79]. Our GGT and LDH values in the colostrum were slightly larger than data reported by Belkasmi et al. [34]. In agreement with Massimini et al. [13], lamb’s GGT level showed a great variability in the newborns, while LDH was higher than the ones proposed by Bornéz et al. [82]. However, this is not particularly surprising if we consider that our results assessed GGT and LDH value variations in time.

Our study provided evidence of the correlation between IgG concentration and enzyme activity levels in sheep’s blood and colostrum. The greatest IgG-GGT correlation was found in lamb’s serum and colostrum. Increased GGT expression is related to the high levels of colostral IgG and milk protein [26,27]. The GGT activity gradually increases during pregnancy and first lactation period [83]. At birth, lambs showed GGT levels similar to adults, and after the assumption of colostrum GGT greatly increased in blood serum [79,80,81,82,83,84]. Moreover, a positive correlation between serum GGT and IgG levels was shown in blood samples collected from lambs within 24 h of birth [22,23,24,25,26,27,28,29]. Our findings confirm that the GGT is the most reliable marker to estimate the passive immunity transfer through the colostrum [19,20,21,22,23,24,25,26,27,28,29,30].

Concerning LDH analysis, the results showed that GGT-LDH correlation was always moderate both in lamb’s serum and in the colostrum. Although few papers described a correlation between IgG and LDH in the colostrum, our study showed a highly significant moderate correlation. As previously suggested, this outcome can be explained as an indicator of udder health, and a suitable index for the IgG concentration in milk [85,86]. GGT is confirmed as an indicator of colostrum quality and passive immunity transfer, while the LDH relationship with IgG concentration needs to be studied further. The evaluation of enzyme activity in the colostrum represents a less invasive practice compared to the same analysis of blood serum in newborns. In addition, this analysis is more cost-effective than IgG evaluation.

Our findings considered a small sample size, as witnessed by the great variability of the data. The experimental trial was carried out in pens adapted for the breeding of only 20 animals, in accordance with the sheep welfare guidelines [86]. Beyond that, we also excluded ewes and lambs with clinical symptoms or complicated labor from the study to minimize confounding factors in the analysis. In light of these preliminary results, further investigations, testing different levels of hazelnut skin inclusion and considering a larger number of animals, are needed to determine the range of immunoglobulin and enzyme activity levels under different dietary conditions more precisely.

## 5. Conclusions

Our work demonstrated that hazelnut skin affects the IgG concentration in ewe’s colostrum, with an increased antibody coverage in lambs and better proactive immune defense against neonatal infectious diseases. The inclusion of hazelnut skin in ruminant feeding represents an interesting source of bioactive compounds with antioxidant activity that affords a paramount opportunity to benefit the health of farmed animals. The study suggests that the high content of MUFA, PUFA, and the oleic acid of hazelnut skin could ameliorate the IgG concentration in the colostrum, thus improving passive immunity transfer to newborns. Our findings confirm that IgG levels may be indirectly measured by GGT in both the blood and colostrum, and LDH evaluation could predict IgG concentrations in the colostrum. Moreover, our findings provide useful information to the feeding industry for new nutritional strategies by promoting a circular economy in the livestock system.

## Figures and Tables

**Table 1:** Ingredients and proximate composition of the experimental diets for gestation and lactation periods.

		Gestation		Lactation	
		CTR	HZN	CTR	HZN
		Ingredients (g/kg DM)			
Hay		664	562	719	637
Experimental concentrates ^1^					
	Barley	110	111	89	90
	Maize	109	111	89	90
	Soybean meal (44% CP)	56	57	45	46
	Rumen-protected fat	18	-	15	-
	Hazelnut skin	-	116	-	94
		Proximate composition (g/kg DM, unless otherwise stated)			
DM (g/kg)		889	891	889	890
Ash		61	53	71	65
CP		116	121	111	115
RDP (% CP)		66.5	60.0	66.6	61.0
EE		35	56	31	49
NDF		533	519	564	554
ADF		306	314	326	323
ADL		51	74	53	72
NSC ^2^		248	243	222	218
NE_L_ (MJ/kg DM)		6.1	6.3	5.9	6.1

CTR = control diet; HZN = hazelnut skin diet; DM = dry matter; CP = crude protein; RDP = rumen degradable protein; EE = ether extract; NDF = neutral detergent fiber; ADF = acid detergent fiber; ADL = acid detergent lignin; NSC = nonstructural carbohydrates; NE_L_ = net energy for lactation. ^1^ Both experimental concentrates also included (g/kg DM): calcium carbonate (19.0), sodium chloride (9.7), precipitated dicalcium phosphate dihydrate (4.8), sodium bicarbonate (2.9), magnesium oxide (1.9), and mineral-vitamin premix (4.8). ^2^ Calculated as 1000 − (NDF + CP + EE + ash).

**Table 2:** Dry matter and main fatty acids intake (g/d) of ewes fed CTR and HZN diets during gestation and lactation periods.

	Gestation		Effects		Lactation		Effects	
	CTR	HZN	DT	SD	CTR	HZN	DT	SD
DMI (kg/d)	1.54	1.51	ns	ns	1.89	1.86	ns	ns
C16:0	12.95	6.55	***	ns	13.57	7.20	***	ns
C18:0	1.41	1.39	***	ns	1.51	1.49	***	ns
C18:1*c*9 (OA)	10.17	37.50	***	ns	10.33	37.66	***	ns
C18:2 *c*6*c*9*c*12 (LA)	10.93	13.54	***	ns	11.43	14.04	***	ns
C18:3 *c*9*c*12*c*15 (ALA)	2.62	2.28	***	ns	3.30	2.96	***	ns
∑SFA	15.96	9.12	***	ns	17.00	10.16	***	ns
∑MUFA	10.70	38.67	***	ns	10.93	38.90	***	ns
∑PUFA	13.69	15.93	***	ns	14.90	17.15	***	ns

CTR = control diet; HZN = hazelnut skin diet; DT = dietary treatment; SD = sampling date; DMI = dry matter intake; OA = oleic acid; LA = linoleic acid; ALA = α-linolenic acid; SFA = saturated fatty acid; MUFA = monounsaturated fatty acid; PUFA = polyunsaturated fatty acid. Probability: *** *p* < 0.001; ns = not significant (*p* > 0.05). The effect of interaction between dietary treatment and sampling date (DT × SD) was not significant; therefore, significance is only presented for the main effects.

**Table 3:** Linear mixed effect models (LMMs) for sheep, lambs and colostrum IgG concentration. Estimates, standard errors, t-values, as well as the significant level is provided for each model.

	Sheep ^1^ (pseudoR^2^ = 0.04)		Lambs ^2^ (pseudoR^2^ = 0.77)		Colostrum ^1^ (pseudoR^2^ = 0.75)	
IgG (g/L)	Intercept	HZN	Intercept	HZN	Intercept	HZN
Estimate	17.2042	−1.0833	15.228	2.503	29.078	12.947
Standard Error	0.5671	0.8021	3.833	1.036	17.898	5.286
t-value	30.335	−1.351	3.973	2.415	3.139	43.999
*p*-value	<2 × 10^−16^	0.183	0.0154	0.019	1.625	0.0184

HZN = hazelnut skin diet. ^1^ Total number of samples equal to 48 (eight ewes × two groups × three sampling dates). ^2^ Total number of samples equal to 64 (eight lambs × two groups × four sampling dates).

## Data Availability

Not applicable.

## Supplementary Materials

The following supporting information can be downloaded at https://www.mdpi.com/article/10.3390/ani12223220/s1, Table S1: Proximate composition and fatty acid profile of experimental feedstuffs; Table S2: IgG concentration (g/L) in sheep and lamb’s blood serum and colostrum during the whole sampling period.

## References

[1] Lanza M., Bella M., Priolo A., Barbagallo D., Galofaro V., Landi C., Pennisi P. (2006). Lamb meat quality as affected by a natural or artificial milk feeding regime. Meat Sci..

[2] Hernández-Castellano L.E., Suárez-Trujillo A., Martell-Jaizme D., Cugno G., Argüello A., Castro N. (2015). The effect of colostrum period management on BW and immune system in lambs: From birth to weaning. Animal.

[3] Cabral R.G., Chapman C.E., Aragona K.M., Clark E., Lunak M., Erickson P.S. (2016). Predicting colostrum quality from performance in the previous lactation and environmental changes. J. Dairy Sci..

[4] Loste A., Ramos J.J., Fernández A., Ferrer L.M., Lacasta D., Verde M.T., Marca M.C., Ortín A. (2008). Effect of colostrum treated by heat on immunological parameters in newborn lambs. Livest. Sci..

[5] Weaver D.M., Tyler J.W., VanMetre D.C., Hostetler D.E., Barrington G.M. (2000). Passive Transfer of Colostral Immunoglobulins in Calves. J. Vet. Intern. Med..

[6] Ahmad R., Khan A., Javed M.T., Hussain I. (2000). The level of immunoglobulins in relation to neonatal lamb mortality in Pak-Karakul sheep. Vet. Arh..

[7] Hernández-Castellano L., Wall S.K., Stephan R., Corti S., Bruckmaier R. (2017). Milk somatic cell count, lactate dehydrogenase activity, and immunoglobulin G concentration associated with mastitis caused by different pathogens: A field study. Schweiz. Arch. Tierheilkd..

[8] Braun J.P., Trumel C., Bézille P. (2010). Clinical biochemistry in sheep: A selected review. Small Rumin. Res..

[9] Conneely M., Berry D.P., Sayers R., Murphy J.P., Lorenz I., Doherty M.L., Kennedy E. (2013). Factors associated with the concentration of immunoglobulin G in the colostrum of dairy cows. Animal.

[10] Ahmadi M., Boldura O., Milovanov C., Dronca D., Mircu C., Hutu I., Popescu S., Padeanu I., Tulcan C. (2016). Colostrum from Different Animal Species–A Product for Health Status Enhancement. Bull. Univ. Agric. Sci. Vet. Med. Cluj-Napoca.

[11] Torres-Rovira L., Pesantez-Pacheco J.-L., Hernandez F., Elvira-Partida L., Perez-Solana M.-L., Gonzalez-Martin J.-V., Gonzalez-Bulnes A., Astiz S. (2017). Identification of factors affecting colostrum quality of dairy Lacaune ewes assessed with the Brix refractometer. J. Dairy Res..

[12] Zarei S., Ghorbani G.R., Khorvash M., Martin O., Mahdavi A.H., Riasi A. (2017). The Impact of Season, Parity, and Volume of Colostrum on Holstein Dairy Cows Colostrum Composition. Agric. Sci..

[13] Massimini G., Peli A., Boari A., Britti D. (2006). Evaluation of assay procedures for prediction of passive transfer status in lambs. Am. J. Vet. Res..

[14] Tarquino C.F., Flaiban K.M.C., Lisboa J.A.N. (2011). Transferência de imunidade passiva em cordeiros de corte manejados extensivamente em clima tropical. Braz. J. Vet. Res..

[15] Alves A.C., Alves N.G., Ascari I.J., Junquinera F.B., Coutinho A.S., Lima R.R., Pérez J.R.O., De Paula S.O., Furusho-Garcia I.F., Abreu L.R. (2015). Colostrum composition of Santa Inês sheep and passive transfer of immunity to lambs. J. Dairy Sci..

[16] Highland M.A., Berglund A.K., Knowles D.P. (2017). Passive transfer in domestic and bighorn lambs total IgG in ewe sera and colostrum and serum IgG kinetics in lambs following colostrum ingestion are similar in domestic sheep and bighorn sheep (*Ovis aries* and *Ovis canadensis*). Sheep Goat Res. J..

[17] Kessler E.C., Bruckmaier R.M., Gross J.J. (2019). Immunoglobulin G content and colostrum composition of different goat and sheep breeds in Switzerland and Germany. J. Dairy Sci..

[18] Maden M., Birdane F.M., Altunok V., Dere S. (2004). Serum and colostrum/milk alkaline phosphatase activities in the determination of passive transfer status in healthy lambs. Rev. Med. Vet..

[19] Britti D., Massimini G., Peli A., Luciani A., Boari A. (2005). Evaluation of serum enzyme activities as predictors of passive transfer status in lambs. J. Am. Vet. Med. Assoc..

[20] Hogan I., Doherty M., Fagan J., Kennedy E., Conneely M., Brady P., Ryan C., Lorenz I. (2015). Comparison of rapid laboratory tests for failure of passive transfer in the bovine. Ir. Vet. J..

[21] Gökçe E., Atakişi O. (2018). Interrelationships of Serum and Colostral IgG (Passive Immunity) with Total Protein Concentrations and Health Status in Lambs. Kafkas Univ. Vet. Fak. Derg..

[22] Tessman R.K., Tyler J.W., Parish S.M., Johnson D.L., Gant R.G., Grasseshi H.A. (1997). Use of age and serum gamma-glutamyl-transferase activity to assess passive transfer status in lambs. J. Am. Vet. Med. Assoc..

[23] Tate S.S., Meister A., Najjar V.A. (1981). γ-Glutamyl transpeptidase: Catalytic, structural and functional aspects. The Biological Effects of Glutamic Acid and Its Derivatives.

[24] Hu G., Tuomilehto J., Pukkala E., Hakulinen T., Antikainen R., Vartiainen E., Jousilahti P. (2008). Joint effects of coffee consumption and serum gamma-glutamyltransferase on the risk of liver cancer. Hepatology.

[25] Souza J.F., Longui C.A., Miorin L.A., Sens Y.A. (2008). Gamma-Glutamyltransferase Activity in Chronic Dialysis Patients and Renal Transplant Recipients With Hepatitis C Virus Infection. Transplant. Proc..

[26] Vina J.R., Garcia C., Barber T. (2001). Regulation of amino acid metabolism during lactation. Recent Res. Dev. Nutr..

[27] Pero M.E., Mirabella N., Lombardi P., Squillacioti C., De Luca A., Avallone L. (2006). Gammaglutamyltransferase activity in buffalo mammary tissue during lactation. Anim. Sci..

[28] Lehmann M., Wellnitz O., Bruckmaier R.M. (2013). Concomitant lipopolysaccharide-induced transfer of blood-derived components including immunoglobulins into milk. J. Dairy Sci..

[29] Lombardi P., Avallone L., Pagnini U., D’Angelo D., Bogin E. (2001). Evaluation of Buffalo Colostrum Quality by Estimation of Enzyme Activity Levels. J. Food Prot..

[30] Maden M., Altunok V., Birdane F.M., Aslan V., Nizamlioglu M. (2003). Blood and Colostrum/Milk Serum γ-Glutamyltransferase Activity as a Predictor of Passive Transfer Status in Lambs. J. Vet. Med. Ser. B.

[31] Zarrilli A., Micera E., Lacarpia N., Lombardi P., Pero M.E., Pelagalli A., D’Angelo D., Mattia M., Avallone L. (2003). Evaluation of goat colostrum quality by determining enzyme activity levels. Livest. Prod. Sci..

[32] Zarrilli A., Micera E., Lacarpia N., Lombardi P., Pero M.E., Pelagalli A., D’Angelo D., Mattia M., Avallone L. (2003). Evaluation of ewe colostrum quality by estimation of enzyme activity levels. Rev. Med. Vet..

[33] Aydogdu U., Guzelbektes H. (2019). Effect of colostrum composition on passive calf immunity in primiparous and multiparous dairy cows. Vet. Med..

[34] Belkasmi F., Madani T., Mouffok C., Semara L. (2019). Enzymatic quality of colostrum in Ouled Djellal ewes, Algeria. Biol. Rhythm Res..

[35] Pecka-Kiełb E., Zachwieja A., Wojtas E., Zawadzki W. (2018). Influence of nutrition on the quality of colostrum and milk of ruminants. Mljekarstvo.

[36] Basiricò L., Morera P., Dipasquale D., Tröscher A., Serra A., Mele M., Bernabucci U. (2015). Conjugated linoleic acid isomers strongly improve the redox status of bovine mammary epithelial cells (BME-UV1). J. Dairy Sci..

[37] Abuelo A., Hernández J., Benedito J.L., Castillo C. (2019). Redox Biology in Transition Periods of Dairy Cattle: Role in the Health of Periparturient and Neonatal Animals. Antioxidants.

[38] Serra V., Salvatori G., Pastorelli G. (2021). Dietary Polyphenol Supplementation in Food Producing Animals: Effects on the Quality of Derived Products. Animals.

[39] Kasapidou E., Sossidou E., Mitlianga P. (2015). Fruit and Vegetable Co-Products as Functional Feed Ingredients in Farm Animal Nutrition for Improved Product Quality. Agriculture.

[40] Mirabella N., Castellani V., Sala S. (2014). Current options for the valorization of food manufacturing waste: A review. J. Clean. Prod..

[41] Schieber A., Stintzing F.C., Carle R. (2001). By-products of plant food processing as a source of functional compounds—recent developments. Trends Food Sci. Technol..

[42] Liso G., Palmieri A., Pirazzoli C., Moriello M.S. (2017). Prospettive e opportunità in Italia per un’efficiente filiera corilicola. Suppl. Terra Vita.

[43] Caccamo M., Valenti B., Luciano G., Priolo A., Rapisarda T., Belvedere G., Marino V.M., Esposto S., Taticchi A., Servili M. (2019). Hazelnut as Ingredient in Dairy Sheep Diet: Effect on Sensory and Volatile Profile of Cheese. Front. Nutr..

[44] Campione A., Natalello A., Valenti B., Luciano G., Rufino-Moya P.J., Avondo M., Morbidini L., Pomente C., Krol B., Wilk M. (2020). Effect of Feeding Hazelnut Skin on Animal Performance, Milk Quality, and Rumen Fatty Acids in Lactating Ewes. Animals.

[45] Renna M., Lussiana C., Malfatto V., Gerbelle M., Turille G., Medana C., Ghirardello D., Mimosi A., Cornale P. (2020). Evaluating the Suitability of Hazelnut Skin as a Feed Ingredient in the Diet of Dairy Cows. Animals.

[46] Pereira Neves A. (2016). Desempenho de Ovelhas Pantaneiras Submetidas à Sincronização de Estro e Suplementação Nutricional de Curto Prazo Antes da Estação Reprodutiva. Master’s Thesis.

[47] Spezzigu A., Sale S., Bua S. (2017). Controllo ormonale della dinamica follicolare nella pecora: Protocolli di sincronizzazione dei calori. Summa.

[48] National Research Council (NRC) (2007). Nutrient Requirements of Small Ruminants: Sheep, Goats, Cervids, and New World Camelids.

[49] Association of Official Analytical Chemists (AOAC) (2000). Official Methods of Analysis.

[50] Association of Official Analytical Chemists (AOAC) (2013). Official Methods of Analysis.

[51] Van Soest P.J., Robertson J.B., Lewis B.A. (1991). Methods for dietary fiber, neutral detergent fiber, and nonstarch polysaccharides in relation to animal nutrition. J. Dairy Sci..

[52] Licitra G., Hernandez T.M., Van Soest P.J. (1996). Standardization of procedures for nitrogen fractionation of ruminant feeds. Anim. Feed Sci. Technol..

[53] Nutrient Research Council (NRC), Board on Agriculture and Natural Resources, Subcommittee on Dairy Cattle Nutrition, Committee on Animal Nutrition (2001). Nutrient Requirements of Dairy Cattle: Seventh Revised Edition.

[54] Alves S.P., Cabrita A.R.J., Fonseca A.J.M., Bessa R.J.B. (2008). Improved method for fatty acid analysis in herbage based on direct transesterification followed by solid-phase extraction. J. Chromatogr. A.

[55] Cornale P., Renna M., Lussiana C., Bigi D., Chessa S., Mimosi A. (2014). The Grey Goat of Lanzo Valleys (Fiurinà): Breed characteristics, genetic diversity, and quantitative-qualitative milk traits. Small Rumin. Res..

[56] Iussig G., Renna M., Gorlier A., Lonati M., Lussiana C., Battaglini L.M., Lombardi G. (2015). Browsing ratio, species intake, and milk fatty acid composition of goats foraging on alpine open grassland and grazable forestland. Small Rumin. Res..

[57] Enri S.R., Probo M., Renna M., Caro E., Lussiana C., Battaglini L.M., Lombardi G., Lonati M. (2020). Temporal variations in leaf traits, chemical composition and in vitro true digestibility of four temperate fodder tree species. Anim. Prod. Sci..

[58] Hunter A.G., Reneau J.K., Williams J.B. (1977). Factors Affecting IgG Concentration in Day-Old Lambs. J. Anim. Sci..

[59] Amadori M., Archetti I.L. (2002). La valutazione del benessere nella specie bovina. Fondazione Iniziative Zooprofilattiche e Zootecniche.

[60] Gornall A.G., Bardawill C.J., David M.M. (1949). Determination of serum proteins by means of the biuret reaction. J. Biol. Chem..

[61] Wickham H. (2016). Ggplot2: Elegant Graphics for Data Analysis.

[62] Bates D., Mächler M., Bolker B., Walker S. (2015). Fitting linear mixed-effects models using lme4. J. Stat. Softw..

[63] Kuznetsova A., Brockhoff P.B., Christensen R.H.B. (2017). lmerTest Package: Tests in linear mixed effects models. J. Stat. Softw..

[64] Akaike H., Petrov B.N., Csaki F. (1973). Information Theory and an Extension of the Maximum Likelihood Principle. 2nd International Symposium on Information Theory, Proceedings of the 2nd International Symposium on Information Theory, Tsahkadsor, Armenia, USSR, 2–8 September 1971.

[65] Sarkar D. (2008). Lattice: Multivariate Data Visualisation with R.

[66] Weiss W.P., Spears J.W. (2006). Vitamin and trace mineral effects on immune function of ruminants. Ruminant Physiology.

[67] Hatfield P.G., Daniels J.T., Kott R.W., Burgess D.E., Evans T.J. (2000). Role of supplemental vitamin E in lamb survival and production: A review. J. Anim. Sci..

[68] Bondo T., Jensen S.K. (2010). Administration of RRR-α-tocopherol to pregnant mares stimulates maternal IgG and IgM production in colostrum and enhances vitamin E and IgM status in foals. J. Anim. Physiol. Anim. Nutr..

[69] Wang L., Xu X., Su G., Shi B., Shan A. (2016). High concentration of vitamin E supplementation in sow diet during the last week of gestation and lactation affects the immunological variables and antioxidative parameters in piglets. J. Dairy Res..

[70] Żarczyńska K., Samardžija M., Sobiech P. (2019). Influence of selenium administration to dry cows on selected biochemical and immune parameters of their offspring. Reprod. Domest. Anim..

[71] Jensen S.K., Lauridsen C. (2007). α-Tocopherol Stereoisomers. Vitam. Horm..

[72] Sterndale S., Broomfield S., Currie A., Hancock S., Kearney G.A., Lei J., Liu S., Lockwood A., Scanlan V., Smith G. (2018). Supplementation of Merino ewes with vitamin E plus selenium increases α-tocopherol and selenium concentrations in plasma of the lamb but does not improve their immune function. Animal.

[73] Lipiński K., Mazur-Kuśnirek M., Antoszkiewicz Z., Purwin C. (2017). Polyphenols in Monogastric Nutrition—A Review. Ann. Anim. Sci..

[74] Vasta V., Daghio M., Cappucci A., Buccioni A., Serra A., Viti C., Mele M. (2019). Invited review: Plant polyphenols and rumen microbiota responsible for fatty acid biohydrogenation, fiber digestion, and methane emission: Experimental evidence and methodological approaches. J. Dairy Sci..

[75] Choi J., Kim W.K. (2020). Dietary Application of Tannins as a Potential Mitigation Strategy for Current Challenges in Poultry Production: A Review. Animals.

[76] Caprarulo V., Giromini C., Rossi L. (2020). Review: Chestnut and quebracho tannins in pig nutrition: The effects on performance and intestinal health. Animal.

[77] Prodanović R., Nedić S., Simeunović P., Borozan S., Nedić S., Bojkovski J., Kirovski D., Vujanac I. (2021). Effects of chestnut tannins supplementation of prepartum moderate yielding dairy cows on metabolic health, antioxidant and colostrum indices. Ann. Anim. Sci..

[78] Vela C.M. (2011). Avaliação da Absorção Colostral em Neonatos Ovinos da raça Bergamácia. Master’s Thesis.

[79] Pugh D.G., Braid A.N. (2018). Reference intervals and conversions. Sheep and Goat Medicine.

[80] Zhu H., Zhao X., Chen S., Tan W., Han R., Qi Y., Huang D., Yang Y. (2021). Evaluation of colostrum bioactive protein transfer and blood metabolic traits in neonatal lambs in the first 24 hours of life. J. Dairy Sci..

[81] Vatankhan M. (2013). Relationship between immunoglobulin concentrations in the ewe’s serum and colostrum, and lamb’s serum in Lori-Bakhtiari Sheep. Iran. J. Appl. Anim. Sci..

[82] Bórnez R., Linares M.B., Vergara H. (2009). Haematological, hormonal and biochemical blood parameters in lamb: Effect of age and blood sampling time. Livest. Sci..

[83] Cestaro A. (2008). Meccanismi di Trasferimento Della Componente Proteica Colostrale Nella Specie Bufalina. Ph.D. Thesis.

[84] Pauli J.V. (1983). Colostral transfer of gamma glutamyl transferase in lambs. N. Z. Vet. J..

[85] Chagunda M.G., Larsen T., Bjerring M., Ingvartsen K.L. (2006). L-lactate dehydrogenase and N-acetyl-β-D-glucosaminidase activities in bovine milk as indicators of non-specific mastitis. J. Dairy Res..

[86] Experimental Zooprofilactic Institute of Lombardy and Emilia Romagna Bruno Ubertini (IZSLERBU) (2021). Valutazione del Benessere Animale nell’Allevamento degli Ovini e dei Caprini: Manuale Esplicativo Controllo Ufficiale.

