# Supplementary material for: Hazelnut Skin in Ewes’ Diet: Effects on Colostrum Immunoglobulin G and Passive Transfer of Immunity to the Lambs
Source: Animals (Basel). 2022 Nov 21;12(22):3220. doi: 10.3390/ani12223220 (PMC9686705; doi:10.3390/ani12223220)
Supplement: Supplementary file 1 [file animals-12-03220-s001.zip › animals-2018922-Table S1.pdf]

Table S1. Proximate composition and fatty acid profile of experimental feedstuffs.

	Hay	Barley	Maize	Soybean meal	Rumen protected fat	Hazelnut skin
	Proximate composition (g/kg DM, unless otherwise stated)					
DM (g/kg)	889	881	880	900	980	916
Ash	83	25	15	67	143	25
CP	89	128	103	482	-	112
RDP (% CP)	66.8	80.0	35.1	72.0	-	8.0
EE	15	21	42	12	857	327
NDF	702	218	101	158	-	501
ADF	413	85	36	106	-	422
ADL	63	16	10	78	-	245
NSC [1]	111	608	739	281	-	35
NE_L (MJ/kg DM)	4.8	8.1	8.9	8.2	19.6	8.8
	Fatty acid (g/kg DM)					
C10:0	0.45	0.45	0.39	0.72	0.41	n.d.
C12:0	n.d.	n.d.	n.d.	n.d.	3.17	0.07
C14:0	0.09	0.09	0.02	0.02	7.79	0.14
C15:0 [2]	0.06	0.03	0.01	0.02	0.19	0.03
C16:0	1.85	6.15	5.42	3.60	284.56	13.86
C16:1 *t*3	0.09	n.d.	n.d.	n.d.	n.d.	n.d.
C17:0*aiso*	0.01	n.d.	n.d.	n.d.	0.14	0.07
C16:1 *c*9	0.03	0.03	0.05	0.02	0.98	0.39
C17:1 *c*9	0.01	0.01	0.02	0.01	0.25	0.14
C18:0	0.28	0.55	0.91	1.01	25.44	4.42
C18:1 *t*	0.01	0.01	0.02	0.01	1.89	n.d.
C18:1 *c*9	0.45	3.68	11.30	2.47	225.80	189.90
C18:1 *c*11	0.04	0.17	0.28	0.31	n.d.	3.58
C18:2 *c*9*c*12	1.42	14.64	23.53	11.65	49.61	24.19
C20:0	0.09	0.06	0.14	0.04	1.98	0.26
C18:3 *c*6*c*9*c*12	0.02	0.01	0.01	0.01	0.11	n.d.
C20:1 *c*9	0.01	0.16	0.09	0.02	0.78	0.31
C18:3 *c*9*c*12*c*15	1.95	1.30	0.51	1.68	1.64	0.36
C20:2 n6	n.d.	0.02	0.01	0.01	0.02	n.d.
C22:0	0.11	0.05	0.04	0.06	0.30	0.10
C20:5 n3	0.08	0.02	0.04	0.03	0.19	n.d.
Σ SFA	2.97	7.36	6.93	5.47	323.99	18.95
Σ MUFA	0.66	4.07	11.76	2.84	229.71	194.32
Σ PUFA	3.48	15.99	24.09	13.38	51.60	24.55
TFA	7.10	27.43	42.78	21.69	605.30	237.82

* DM = dry matter; CP = crude protein; RDP = rumen degradable proteinEE = ether extract; NDF = neutral detergent fiber; ADF = acid detergent fiber; ADL = acid detergent lignin; NSC = nonstructural carbohydrates; NE_L = net energy for lactation; n.d.=not detected; *c=cis*; *t=trans*; SFA=saturated fatty acids; MUFA=monounsaturated fatty acids; PUFA=polyunsaturated fatty acids; TFA=total fatty acids.

[1] Calculated as 1000 − (NDF + CP + EE + ash).

[2] Coeluted with C14:1
