# Supplementary material for: Hazelnut Skin in Ewes’ Diet: Effects on Colostrum Immunoglobulin G and Passive Transfer of Immunity to the Lambs
Source: Animals (Basel). 2022 Nov 21;12(22):3220. doi: 10.3390/ani12223220 (PMC9686705; doi:10.3390/ani12223220)
Supplement: Supplementary file 1 [file animals-12-03220-s001.zip › animals-2018922-Table S2.pdf]

Table S2. IgG concentration (g/l) in sheep and lamb's blood serum and colostrum during the whole sampling period. Data are expressed as mean ± SD among the time (A = at birth, B = 24h after birth, C = 48h after birth, D = 10 days after birth).

Sample	Group (n = 8)[1]	Sampling time			
		A	B	C	D
Sheep	CTR	16.5±3.2	18.1±4.4	17.1±3.5	n.d.
	HZN	16.0±1.6	16.4±1.7	15.9±2.1	n.d.
Colostrum	CTR	60.4±10.7	19.1±10.4	6.6±4.4	n.d.
	HZN	93.7±35.9	27.6±13.6	4.8±2.4	n.d.
Lambs	CTR	5.2±1.5	22.8±6.9	18.2±6.0	14.7±3.8
	HZN	4.0±1.2	27.3±3.0	22.7±3.6	17.0±3.2

* CTR = control group; HZN = hazelnut skin group; A = at birth; B = 24h after birth; C = 48h after birth; D = 10 days after birth; n.d. = not determined

[1] Total number of samples for sheep and colostrum equal to 48 (8 ewes × 2 groups × 3 sampling dates); total number of samples for lambs equal to 64 (8 lambs × 2 groups × 4 sampling dates).
